# Urine Phthalate Levels and Liver Function in US Adolescents: Analyses of NHANES 2007–2016

**DOI:** 10.3389/fpubh.2022.843971

**Published:** 2022-03-04

**Authors:** Shiting Xiang, Jie Dong, Xun Li, Chao Li

**Affiliations:** ^1^Pediatrics Research Institute of Hunan Province, Hunan Children's Hospital, Changsha, China; ^2^Department of Epidemiology and Medical Statistics, Xiangya School of Public Health, Central South University, Changsha, China

**Keywords:** phthalate, liver, adolescents, NHANES, indicators

## Abstract

**Background:**

Phthalates are non-persistent chemicals with endocrine-disrupting abilities widely used in a variety of consumer products. Evidence for the effects of phthalate exposure on liver function in adolescents is lacking.

**Methods:**

Data were analyzed from the combined 2007–2016 National Health and Nutrition Examination Survey (NHANES). Ultimately, a total of 1,650 adolescents aged 12–19 years were selected as the samples. Weighted linear regression was used to investigate the effects of urinary phthalate metabolites on liver function indexes.

**Results:**

Weighted Linear regression models showed that MCOP was negatively associated with TBIL (β = −0.0435, *P*_FDR_ = 0.007), ΣDEHP (β = −0.0453, *P*_FDR_ = 0.003) and MCOP (β = −0.0379, *P*_FDR_ = 0.006) were negatively correlated with ALB, while MCPP was positively correlated with ALB (β = 0.0339, *P*_FDR_ = 0.024), and MCOP was negatively correlated with TP (β = −0.0551; *P*_FDR_ = 0.004).

**Conclusions:**

Phthalate metabolites were significantly but weakly associated with changes in liver function indicators among US adolescents. Future work should further examine these relationships.

## Background

Phthalates, known as plasticizers, are non-persistent chemicals with endocrine-disrupting abilities widely used in a variety of consumer products (1). High molecular weight phthalates, including di-(2-ethylhexyl) phthalate (DEHP) and di-isononyl phthalate (DiNP), are used primarily as plasticizer for polyvinyl chloride, building and construction materials, and several categories of toys (such as plastic books, ball, doll, and cartoon characters). Low molecular weight phthalates, including di-butyl phthalate (DBP) and diethyl phthalate (DEP), are used primarily as fragrance ingredients in cosmetics, home, and personal care products (2, 3). As phthalates are usually bound to polymers by non-chemical bonds, they are often constantly released from plastic products into the surrounding environment, resulting in food, water, or air pollution (4). Human are exposed to large amounts of phthalates through dietary, inhalation and skin contact (4).

Liver diseases such as non-alcoholic liver disease, alcoholic liver disease and viral hepatitis are major causes of illness and death worldwide. Approximately 2 million people die from it every year in the world (5). Although vaccination and new drugs will reduce the burden of viral-related liver disease, non-alcoholic liver disease continues to rise in general population adolescents (6). In addition to alcohol, viruses, genetics, and unhealthy lifestyles, studies have found that environmental chemicals may play a role in abnormal liver function in adolescents (7).

Liver plays an important role in the detoxification of phthalates (8). The hepatotoxicity of phthalates has been demonstrated in animal models such as mice, zebrafish, and quail (9–11). Phthalate concentrations have been adversely associated with indicators of liver function in adulthood (12), but few studies have examined associations between phthalate exposure and liver function in youth. Changes in liver function are a long-term process of liver injury, early prevention and intervention can reduce the incidence of liver disease in adults.

Therefore, in the present study, we aimed to examine the association between phthalate exposure and indicators of liver function using a nationally representative sample of adolescents aged 12–19 years in the United States.

## Methods

### Study Population

National Health and Nutrition Examination Survey (NHANES) is a cross-sectional, nationally representative survey in the United States conducted annually by CDC's National Center for Health Statistics (CDC/NCHS). A detailed description of the study design can be found elsewhere (13). The survey uses a multistage stratified probability sample based on selected counties, blocks, households, and persons within households. Survey interviews were conducted in participants' homes by well-trained professionals, while extensive physical examinations, including blood and urine collection, were conducted at mobile exam centers.

The present analysis included five waves of the NHANES from 2007 to 2016, which were publicly shared and downloaded from the CDC official website and combined according to the NHANES tutorials. The 6,598 participants were between the ages of 12 and 19. A one-third subsample were tested for phthalates (*n* = 2,076). We excluded subjects who were serologically positive for hepatitis B virus or hepatitis C virus and did not have complete records, including liver function tests and covariates. Finally, a total of 1,650 adolescents were selected as final samples.

### Liver Function Measure Outcomes

Fasting blood samples were collected in NHANES participants aged 12 years and older at a mobile examination center. The samples were refrigerated and transported to the central laboratory for analysis of serum liver function indicators using the Beckman Coulter DxC800 Synchron clinical system (14).

The liver is rich in alanine aminotransferase (ALT) and aspartate aminotransferase (AST). Serum levels of these two enzymes rise when hepatocytes necrosis or liver cell membrane damage (15). AST/ALT ratio is used for differential diagnosis of acute and chronic liver diseases. The liver is the only place where albumin (ALB) is synthesized. When liver function is impaired, serum albumin (ALB), and total protein (TP) levels decrease (16). Alkaline phosphotase (ALP) and Gamma glutamyl transferase (GGT) are markers of cholestasis (17). The liver has the functions of uptake, combination, and excretion of bilirubin metabolism. The disorder of one or more functions can lead to the increase of total bilirubin (TBIL) (18).

### Measurement of Phthalate

Phthalate metabolites were measured in spot urine samples from a third of study subjects randomly selected from participants 6 years of age and older. The collected samples were frozen at −20°C and then shipped to the CDC's National Center for Environmental Health for analysis. Urine specimens were processed using high performance liquid chromatography-electrospray ionization-tandem mass spectrometry (HPLC-ESI-MS/MS) for the quantitative detection of phthalate metabolites (14).

We selected 12 metabolites tested in all five rounds and excluded phthalate metabolites whose measured values were more than 40% below the detection limit (LOD). The remaining 11 urinary phthalate metabolites used in our study were mono-(carboxyisononyl) phthalate (MCNP), mono-(carboxyisoctyl) phthalate (MCOP), mono-2-ethyl-5-carboxypentyl phthalate (MECPP), mono-n-butyl phthalate (MnBP), mono-(3-carboxypropyl) phthalate (MCPP), mono-ethyl phthalate (MEP), mono-(2-ethyl-5-hydroxyhexyl) phthalate (MEHHP), mono-(2-ethylhexyl phthalate (MEHP), mono-isobutyl phthalate (MiBP), mono-(2-ethyl-5-oxohexyl) phthalate (MEOHP), and mono-benzyl phthalate (MBzP). Phthalate metabolites concentrations below LODs were replaced with LOD divided by the square root of two.

Concentrations of MECPP, MEHHP, MEHP, and MEOHP were divided by their respective molar weight (MW) to obtain the molar equivalent. We summed the molar equivalents of these metabolites and multiplied by the molar weight of MEHP (MW = 278) to obtain ΣDEHP metabolites (19).

### Measurements of Covariates

Covariates were selected as potential confounders by referencing to previous publications (20, 21). Covariates were age, gender, race, education, ratio of family income to poverty (PIR), physical activity, body mass index (BMI), and total daily protein intake. Physical activity was a dichotomous variable, with yes representing moderate or vigorous intensity sports, fitness, or recreational activities in a typical week. BMI was calculated as weight (kg) /height^2^ (m^2^) measured in the physical examination and categorized into three levels: <25 kg/m^2^ (Normal/Underweight), 25 to <30 kg/m^2^ (overweight) and ≥30 kg/m^2^ (obese) (22). Data on total daily protein intake were measured through a 24-h food recall interview.

### Statistical Analysis

Demographic characteristics were reported as percentages. Phthalate metabolite concentrations and liver function levels were described in quartile range. We used urine creatinine to adjust the concentrations of phthalate metabolites in all statistical analyses (23, 24). Creatinine-adjusted phthalate metabolites concentrations and indicators of liver function were natural log-transformed to make them normally distributed. Spearman's coefficients were used to test the pairwise correlations of phthalate metabolite concentrations (Supplementary Table 2). We performed survey-weighted linear regression to assess the associations of the urinary phthalate metabolites with indicators of liver function. Benjamini-Hochberg false discovery rate (FDR) correction was used to adjust *P-*values to adjust for multiple testing.

All models were adjusted for PIR, BMI, age, gender, race, education, physical activity, and total daily protein intake. All analyses were performed using phthalate-specific subsample weight to account for the complex sampling design and non-response of NHANES. Weights for combined NHANES survey cycles were calculated according to NHANES guidelines. All statistical analyses were performed using R 3.5.3. All test values were 2-sided and *P* <0.05 was considered significant.

## Results

### Study Population

Characteristics of the study subjects are shown in Table 1. Of the 1,650 participants, the average age was 15.49 ± 2.266 years, with female subjects accounting for 46.5%. Most of the participants are Non-Hispanic White, 84.4% of the participants had education less than high school, 67.3% had a ratio of family income to poverty >1, 16.1% were obese, and 77.9% were physically active.

**Table 1:** Demographic characteristics for adolescents aged 12–19 years old in NHANES 2007–2016 (*N* = 1,650).

**Basic characteristics**	***N***	**%**
**Age (years)**		
12–14	631	38.2
15–17	618	37.5
18–19	401	24.3
**Gender**		
Male	883	53.5
Female	767	46.5
**Race**		
Non-Hispanic White	485	29.4
Non-Hispanic Black	408	24.7
Mexican American	376	22.8
Other Hispanic	190	11.5
Other/Mixed	191	11.6
**Education**		
Less than high school	1,393	84.4
High School graduate or GED	131	7.9
More than High	126	7.6
**PIR**		
≤ 1	540	32.7
>1	1,110	67.3
**BMI groups**		
Normal/Underweight (<25)	1,042	63.2
Overweight (25 to <30)	342	20.7
Obese (≥30)	266	16.1
**Physical activity**		
No	365	22.1
Yes	1,285	77.9

### Levels of Urinary Phthalate Metabolites and Liver Function Indicators

Descriptive statistics for phthalate metabolites and liver function indicators are presented in Table 2. The detection rates for the 11 phthalate metabolites ranged from 71.90 to 99.90%. The median concentrations of MECPP, MEHHP, MEHP, MEOHP, MCNP, MCOP, MnBP, MCPP, MEP, MiBP, and MBzP were 7.34, 6.82, 5.03, 6.41, 5.42, 7.09, 7.29, 5.39, 8.47, 6.84, and 6.59 μg/mmol Cr, respectively. Spearman correlation analysis showed that except for MCOP and MEP, all of them were significantly correlated (Supplementary Table 2).

**Table 2:** Distribution of urinary phthalate metabolites and indicators of liver function for adolescents aged 12–19 years old in NHANES 2007–2016 (*N* = 1,650).

	**≥LOD%**	**P25**	**P50**	**P75**
**Urinary phthalate metabolites (μg/mmol Cr)**				
MECPP	99.90	6.77	7.34	7.99
MEHHP	99.60	6.26	6.82	7.50
MEHP	71.90	4.39	5.03	5.73
MEOHP	99.70	5.82	6.41	7.03
MCNP	98.00	4.95	5.42	6.01
MCOP	99.60	6.27	7.09	8.04
MnBP	98.80	6.74	7.29	7.79
MCPP	93.10	4.74	5.39	6.06
MEP	99.90	7.66	8.47	9.35
MiBP	99.40	6.33	6.84	7.31
MBzP	99.00	5.98	6.59	7.27
**Liver function**				
ALT (IU/L)	100.00	14.00	17.00	21.00
AST (IU/L)	100.00	19.00	22.00	26.00
GGT(U/L)	100.00	11.00	13.00	18.00
ALP (IU/L)	100.00	69.00	96.00	170.00
TBIL (mg/dL)	100.00	0.50	0.60	0.80
ALB (g/dL)	100.00	4.30	4.50	4.70
TP (g/dL)	100.00	7.00	7.20	7.50
AST/ALT	100.00	1.09	1.33	1.57

### Survey-Weighted Liner Regression Analyses

The results of survey-weighted linear regression are shown in Table 3. MCOP was negatively associated with TBIL (β = −0.0435, *P*_FDR_ = 0.007). ΣDEHP (β = −0.0453, *P*_FDR_ = 0.003) and MCOP (β = −0.0379, *P*_FDR_ = 0.006) were negatively correlated with ALB, while MCPP was positively correlated with ALB (β = 0.0339, *P*_FDR_ = 0.024). MCOP was negatively correlated with TP (β = −0.0551, *P*_FDR_ = 0.004).No significant linear relationships were found between ALT, AST, GGT, ALP, and ALT/AST with phthalate metabolites.

**Table 3:** Association between log-transformed phthalate metabolites and indicators of liver function for adolescents aged 12–19 years old in NHANES 2007–2016 (*N* = 1,650).

	**β (95% CI)**	***P*** **-value**	***P*** **_FDR_**
**ALT**			
∑DEHP^a^	−0.3339 (−0.7630 0.0952)	0.437	0.863
MCNP	0.2414 (0.3108, 0.7936)	0.662	0.887
MCOP	−0.2438 (−0.8074, 0.3198)	0.665	0.887
MnBP	−0.5485 (−1.2658, 0.1688)	0.445	0.863
MCPP	−0.1163 (−0.5285, 0.2959)	0.778	0.916
MEP	0.1591 (−0.1512, 0.4694)	0.608	0.887
MiBP	0.0998 (−0.7522, 0.9518)	0.907	0.936
MBzP	0.5276 (−0.1920, 1.2472)	0.464	0.873
**AST**			
∑DEHP^a^	0.0460 (−0.2511, 0.3431)	0.877	0.920
MCNP	0.0793 (−0.3238, 0.4842)	0.844	0.916
MCOP	−0.4631 (−0.8743, −0.0519)	0.260	0.693
MnBP	−0.6381 (−1.6631, 0.3869)	0.534	0.877
MCPP	0.1178 (−0.2362, 0.4718)	0.739	0.916
MEP	0.2225 (0.0047, 0.4403)	0.307	0.728
MiBP	1.7819 (−0.0886, 3.6524)	0.341	0.753
MBzP	−0.3969 (−1.3026, 0.5088)	0.661	0.877
**GGT**			
∑DEHP^a^	−0.2084 (−0.5071, 0.0903)	0.485	0.887
MCNP	0.1705 (−0.1851, 0.5261)	0.632	0.887
MCOP	0.1373 (−0.2632, 0.5378)	0.732	0.916
MnBP	1.1198 (0.5187, 1.7209)	0.063	0.310
MCPP	−0.5597 (−0.8166, −0.3028)	0.030	0.192
MEP	−0.1097 (−0.3595, 0.1401)	0.661	0.887
MiBP	−0.7511 (−1.1897, −0.3125)	0.087	0.352
MBzP	−0.0995 (−0.3384, 0.5374)	0.820	0.916
**ALP**			
∑DEHP^a^	1.2759 (−0.8193, 3.3711)	0.543	0.887
MCNP	4.6037 (1.4705, 7.7369)	0.142	0.488
MCOP	−1.4999 (−3.7503, −0.7505)	0.508	0.887
MnBP	−0.8977 (−4.6925, 2.8971)	0.813	0.816
MCPP	−0.7931 (−3.6079, 2.0217)	0.778	0.816
MEP	−0.2738 (−1.8363, 1.2887)	0.861	0.918
MiBP	6.3493 (3.1975, 9.5013)	0.044	0.256
MBzP	3.1292 (0.5445, 5.7139)	0.226	0.629
**TBIL**			
∑DEHP^a^	−0.0197 (−0.0330, −0.0064)	0.140	0.488
MCNP	0.0153 (0.0005, 0.0301)	0.301	0.728
MCOP	−0.0435 (−0.0557, −0.0313)	**<0.001**	**0.007**
MnBP	0.0273 (0.0086 0.0460)	0.145	0.488
MCPP	0.0466 (0.0300, 0.0632)	0.005	0.053
MEP	0.0159 (0.0075, 0.0243)	0.058	0.309
MiBP	−0.0381(−0.0537, −0.0225)	0.015	0.120
MBzP	0.0075 (−0.0050, 0.0200)	0.549	0.887
**ALB**			
∑DEHP^a^	−0.0453 (−0.0564, −0.0342)	**<0.001**	**0.003**
MCNP	−0.0026 (−0.0145, 0.0093)	0.827	0.916
MCOP	−0.0379 (−0.0483, −0.0275)	**<0.001**	**0.006**
MnBP	−0.0151 (−0.0294, −0.0008)	0.298	0.728
MCPP	0.0339 (0.0230, 0.0448)	**0.0019**	**0.024**
MEP	−0.0183 (−0.0262, −0.0114)	0.0082	0.075
MiBP	0.0006 (−0.0140, 0.0128)	0.966	0.966
MBzP	−0.0149 (−0.0269, −0.0029)	0.215	0.625
**TP**			
∑DEHP^a^	−0.0202 (−0.0351, −0.0053)	0.176	0.536
MCNP	0.0017 (−0.0162, 0.0196)	0.925	0.940
MCOP	−0.0551 (−0.0694, −0.0408)	**<0.001**	**0.004**
MnBP	−0.0472 (−0.0682, −0.0262)	0.025	0.178
MCPP	0.0036 (−0.0144, 0.0216)	0.839	0.916
MEP	0.0121 (−0.0004, 0.0246)	0.335	0.753
MiBP	0.0108 (−0.0091, 0.0307)	0.588	0.887
MBzP	−0.0120 (−0.0273, 0.0033)	0.432	0.863
**AST/ALT**			
∑DEHP^a^	0.0026 (0.0017, 0.0035)	0.774	0.916
MCNP	−0.0184 (−0.0290, −0.0078)	0.084	0.352
MCOP	−0.0050 (−0.0143, 0.0043)	0.595	0.887
MnBP	0.0070 (−0.0075, 0.0215)	0.630	0.887
MCPP	0.0098 (−0.0014, 0.0210)	0.383	0.817
MEP	0.0022 (−0.0043, 0.0087)	0.731	0.916
MiBP	0.0195 (0.0058, 0.0332)	0.154	0.493
MBzP	−0.0182 (−0.0289, −0.0075)	0.088	0.352

a *ΣDEHP indicates the creatinine corrected molar sum of DEHP metabolites including: MECPP, MEHHP, MEHP, and MEOHP (expressed as MEHP, molecular weight 278). P_FDR_ is the P-value adjusted by the method of Benjamini-Hochberg false discovery rate (FDR) correction to adjust for multiple testing. All models were adjusted for PIR(≤ 1/>1), BMI (<25/25–30/≥30), age (12–14/15–17/18–19 years), gender (male/female), race/ethnicity (Mexican American/Other Hispanic/Non-Hispanic White/Non-Hispanic Black/Other Race), education (Less than high school/ High School graduate or GED/ More than High), physical activity (Yes/No), and total daily protein intake. Values in bold are statistically significant (P_FDR_ < 0.05)*.

## Discussion

In this cross-sectional, population-based analysis of US adolescents aged 12–19, we found significantly but weakly associations between several phthalate metabolites and TBIL, ALB, and TP. We observed null associations between phthalate metabolites and ALT, AST, GGT, ALP, and ALT/AST. To our knowledge, this is the first study examined the association between urine phthalate metabolites with liver function indexes in the adolescents population.

ALT was mainly distributed in liver. AST was mainly distributed in myocardium, followed by liver. Serum ALT can be sharply increased before the onset of clinical symptoms in patients with acute liver injury, while AST is significantly increased in cases of chronic hepatitis, cirrhosis, and liver cancer (15). ALP and GGT are also abundant in liver cells. Serum ALP and GGT are significantly increased when cholestasis caused by cirrhosis, cholelithiasis, and tumor (17). Yu et al. (12) reported that ΣDEHP was positively correlated with ALT, GGT, and ALP, and MBP was positively correlated with AST. Wang et al. (25) reported that ALT, AST, GGT were significantly raised as compared to the controls with increasing plasma DEHP residues. Our study found phthalate exposure was not significantly associated with ALT, AST, GGT, and ALP. There could be several reasons for these differences. First, it may be because our study only included participants aged from 12 to 19 years old, and the other two studies were based on adults. Previous studies on animal reported that the liver toxicity of phthalates was related to dose and time-dependent (8). Second, we used urine creatinine to adjust the concentrations of phthalate metabolites. Although it is an acceptable urine dilution adjustment when measuring non-persistent chemicals, more precise methods for calculating biomarkers should be considered. Finally, we used single-point urine samples instead of 24-h urine samples to measure phthalate exposure, which may also increase the measurement error. Further studies are needed to replicate these findings.

Bilirubin usually increases with excess bilirubin production (such as hemolysis), hepatocyte injury (such as hepatitis, cirrhosis, and fatty liver), or obstructed bile drainage (such as bile duct stones, pancreatic cancer, and bile duct cancer) (18). Previous studies have reported that phthalate exposure is associated with cholestasis (26, 27). However, our study found that MCOP was negatively correlated with TBIL. This negative correlation may be related to the fact that phthalates are thought to be involved in inducing oxidative stress and inflammation, while TBIL is thought to have potent antioxidant properties (28).

Hepatocytes are the main site of protein synthesis. The decrease of serum albumin and total protein levels indicates the gradual decrease of normal hepatocytes and the poor function of hepatocyte protein synthesis (16). Our study found that ΣDEHP and MCOP were negatively correlated with ALB, as well as MCOP and TP. This finding is consistent with previous studies that showed exposure to phthalates can lead to hepatocyte apoptosis and accelerate liver damage (29–31). Our results also showed that MCPP was positively correlated with ALB. We lack the detailed knowledge to explain this positive correlation, additional studies will be required to clarify the mechanistic link between phthalate exposure and ALB.

The main strength of this study is that we included a representative sample of US adolescents and we used the data that had been consolidated for 10 years. To our knowledge, this is the first study that summarized the urine phthalate levels and seven liver function indicators in adolescents. The study provides more evidence for further studies to demonstrate a correlation between phthalate exposures with liver dysfunction.

Our study has several limitations. First, the NHANES data were cross-sectional, which did not allow us to make causal inferences. Therefore, all relationships are related and further prospective research should be done to overcome this methodological limitation. Regardless, this study provides important information regarding how phthalate levels change in association with subclinical changes in liver function indicators in the US adolescents which have not been previously reported. Second, because we had no information about the subjects' alcohol consumption and smoking, we did not control for these underlying variables and only adjusted for covariates such as age, BMI, and sex. Finally, we measured phthalate exposure using a single-spot urine sample from each subject, possibly without taking into account changes in the human body over time. This may prevent us from obtaining a more precise exposure assessment to reduce exposure misclassification.

## Conclusions

Phthalate metabolites were significantly but weakly associated with changes in liver function indicators among US adolescents. Future work should further examine these relationships.

## Data Availability Statement

The datasets presented in this study can be found in online repositories. The names of the repository/repositories and accession number(s) can be found at: https://www.cdc.gov/nchs/nhanes/index.htm.

## Ethics Statement

The studies involving human participants were reviewed and approved by http://www.cdc.gov/nchs/nhanes/irba98.htm. Written informed consent to participate in this study was provided by the participants' legal guardian/next of kin.

## Author Contributions

STX contributed to designing this article, performing the statistical analyses, and drafting the manuscript. CL provided the statistical analyses. XL and JD provided critical revision of the manuscript. All authors read and gave final approval of the version to be published.

## Funding

This work was supported by the Hunan Provincial Natural Science Foundation Youth Foundation (2021JJ40275).

## Conflict of Interest

The authors declare that the research was conducted in the absence of any commercial or financial relationships that could be construed as a potential conflict of interest.

## Publisher's Note

All claims expressed in this article are solely those of the authors and do not necessarily represent those of their affiliated organizations, or those of the publisher, the editors and the reviewers. Any product that may be evaluated in this article, or claim that may be made by its manufacturer, is not guaranteed or endorsed by the publisher.

## Abbreviations

DEHP: di-(2-ethylhexyl) phthalate
DiNP: di-isononyl phthalate
DBP: di-butyl phthalate
DEP: diethyl phthalate
NHANES: National Health and Nutrition Examination Survey
CDC: Centers for Disease Control
NCHS: National Center for Health Statistics
ALT: Alanine aminotransferase
AST: Apartate aminotransferase
ALB: Albumin
TP: Total protein
ALP: Alkaline phosphotase
GGT: Gamma glutamyl transferase
TBIL: total bilirubin
HPLC-ESI-MS/MS: high performance liquid chromatography-electrospray ionization-tandem mass spectrometry
LLOD: Lower limit of detection
MCNP: mono-(carboxyisononyl) phthalate
MCOP: mono-(carboxyisoctyl) phthalate
MECPP: mono-2-ethyl-5-carboxypentyl phthalate
MnBP: mono-n-butyl phthalate
MCPP: mono-(3-carboxypropyl) phthalate
MEP: mono-ethyl phthalate
MEHHP: mono-(2-ethyl-5-hydroxyhexyl) phthalate
MEHP: mono-(2-ethylhexyl phthalate
MiBP: mono-isobutyl phthalate
MEOHP: mono-(2-ethyl-5-oxohexyl) phthalate
MBzP: mono-benzyl phthalate
BMI: Body mass index
PIR: Ratio of family income to poverty
FDR: Benjamini-Hochberg false discovery rate.
