# Supplementary material for: Urine Phthalate Levels and Liver Function in US Adolescents: Analyses of NHANES 2007–2016
Source: Front Public Health. 2022 Mar 4;10:843971. doi: 10.3389/fpubh.2022.843971 (PMC8934389; doi:10.3389/fpubh.2022.843971)
Supplement: Supplementary file 1 [file Data_Sheet_1.docx]

Supplemental file

Table S1. The limit of detection (LOD, in ng/mL) for urinary phthalate metabolites (NHANES 2007–2016).

Table S2. Spearman rank correlation matrix for urinary phthalate metabolites concentrations (μg/mmol Cr) measured in the adolescents (N=1,650), NHANES 2007-2016.

Table S1. The limit of detection (LOD, in ng/mL) for urinary phthalate metabolites (NHANES 2007–2016).

| Analyte | 2007-2008 | 2009-2010 | 2011-2012 | 2013-2014 | 2015-2016 |
| --- | --- | --- | --- | --- | --- |
| MCNP | 0.5 | 0.2 | 0.2 | 0.2 | 0.2 |
| MCOP | 0.7 | 0.2 | 0.2 | 0.3 | 0.3 |
| MECPP | 0.5 | 0.2 | 0.2 | 0.4 | 0.4 |
| MnBP | 0.6 | 0.4 | 0.4 | 0.4 | 0.4 |
| MCPP | 0.2 | 0.2 | 0.2 | 0.4 | 0.4 |
| MEP | 0.462 | 0.462 | 0.6 | 1.2 | 1.2 |
| MEHHP | 0.7 | 0.2 | 0.2 | 0.4 | 0.4 |
| MEHP | 1.1 | 0.5 | 0.5 | 0.8 | 0.8 |
| MiBP | 0.3 | 0.2 | 0.2 | 0.8 | 0.8 |
| MEOHP | 1.848 | 0.2 | 0.2 | 0.2 | 0.2 |
| MBzP | 0.216 | 0.216 | 0.3 | 0.3 | 0.3 |

Table S2 Spearman rank correlation matrix for urinary phthalate metabolites concentrations (μg/mmol Cr) measured in the adolescents (N=1,650), NHANES 2007-2016.

|  | ∑DEHP^a^ | MCNP | MCOP | MnBP | MCPP | MEP | MiBP | MBzP |
| --- | --- | --- | --- | --- | --- | --- | --- | --- |
| ∑DEHP^a^ | 1 |  |  |  |  |  |  |  |
| MCNP | 0.319^**^ | 1 |  |  |  |  |  |  |
| MCOP | 0.216^**^ | 0.625^**^ | 1 |  |  |  |  |  |
| MnBP | 0.497^**^ | 0.095^**^ | 0.046 | 1 |  |  |  |  |
| MCPP | 0.529^**^ | 0.541^**^ | 0.622^**^ | 0.356^**^ | 1 |  |  |  |
| MEP | 0.283^**^ | 0.052^**^ | -0.045 | 0.325^**^ | 0.167^**^ | 1 |  |  |
| MiBP | 0.315^**^ | 0.077^**^ | 0.107^**^ | 0.607^**^ | 0.195^**^ | 0.220^**^ | 1 |  |
| MBzP | 0.397^**^ | 0.062^**^ | 0.081^**^ | 0.585^**^ | 0.309^**^ | 0.182^**^ | 0.393^**^ | 1 |

^**^ P< 0.05
